# Green Recovery and Identification of Antioxidant and Enzyme Inhibitor Molecules from Pisco Grape Pomace by Targeted Effects Analysis Using Thin-Layer Chromatography, Bioassay, and Mass Spectrometry

**DOI:** 10.3390/antiox13111418

**Published:** 2024-11-19

**Authors:** Jacqueline Poblete, Joaquín Fernández-Martínez, Mario Aranda, Issis Quispe-Fuentes

**Affiliations:** 1 Food Engineering Department, Universidad de La Serena, Av. Raúl Bitrán 1305, La Serena 1700000, Chile; j.pobletegalleguillos@gmail.com; 2 Department of Chemistry and Pharmacy, Pontificia Universidad Católica de Chile, Santiago 7810000, Chile

**Keywords:** pisco grape pomace, antioxidants, cyclooxygenase, acetylcholinesterase, quercetin, flavonoids

## Abstract

The search and identification of inhibitory molecules from novel natural sources, such as pisco grape pomace extract obtained by green techniques, may help to develop agents with therapeutic potential that are beneficial to health with fewer adverse effects than drugs. Many drugs act as enzyme inhibitors, decreasing their activity and thus correcting a metabolic imbalance. This study aims to identify bioactive molecules with antioxidant and inhibitory activity over acetylcholinesterase and cyclooxygenase enzymes present in pisco grape pomace green extracts. Bioactive molecules were detected and identified applying directed effect analysis on planar chromatography coupled to mass spectrometry. For the first time, the presence of antioxidant molecules (quercetin-3-O-glucuronide, quercetin-3-O-glucoside, and gallic acid) and inhibitors of acetylcholinesterase (kaempferol-3-O-glucoside) and cyclooxygenase (gallic acid) enzymes are reported in pisco grape pomace. According to the results, grape pomace could be an alternative to develop novel functional foods and nutraceuticals that provide health benefits and, at the same time, generate a circular economy in the industry.

## 1. Introduction

Isolated and biologically active molecules have generated great interest in the agri-food, pharmaceutical, nutraceutical, and cosmetic industries because they can exert a wide range of biological activities (antioxidant, antimicrobial, anti-inflammatory, wound healing, anti-aging, anti-hyperpigmentation, photoprotective, chemopreventive, anticancer, antidiabetic, immunomodulatory, antitumor, and cardioprotective) [1,2]. These molecules are found in plants and fruit, showing a broad spectrum of pharmacological effects and resulting in a reduction in the risk of suffering chronic non-communicable diseases (CNCDs) [3]. CNCDs are the leading cause of death and disability worldwide, with 41 million people currently dying each year, accounting for 71% of all deaths globally [4].

Grape pomace is an interesting potential source of biologically active molecules. It is a byproduct of the pisco industry that is obtained in large quantities and is mainly discarded as waste or used for composting and animal feed. This residue is composed of seeds and skins that have a high content of (poly) phenols with an important antioxidant capacity [5,6,7]. Ecological or green extraction techniques are very promising to extract this kind of molecule because they improve the sustainability of extraction by being safe, effective, and economical. Furthermore, these approaches solve the disadvantages of conventional methods, such as low yields, higher solvent volumes, long times, energy, costs, and others [8]. Additionally, in grape pomace, some phenolic compounds are bound to the cell wall or other constituents, which could reduce the extraction yields. To overcome this issue, some novel techniques like enzyme-assisted extraction (EAE) and pressurized liquid extraction (PLE) have been used. The first degrades plant cell wall components using specific enzymes [9] and the second uses water and organic solvents in combination with elevated temperature and pressure [10,11] to improve extraction yields using green solvents like water and ethanol [9,10].

Searching for and identifying inhibitors from novel matrices can be an alternative to developing novel functional foods, ingredients, nutraceuticals, and agents with therapeutic potential that provide health benefits with fewer adverse effects. Enzymatic inhibition is one of the most relevant therapeutic targets, and this kind of molecule is highly distributed in nature. For example, some naturally occurring acetylcholinesterase inhibitors like galantamine are commercially available to treat Alzheimer’s disease [12]. Some studies have reported the presence of different types of inhibitors in natural products like cherimoya, fig, ginger, and Tunisian sages [3,13,14,15,16]. This inhibitory effect has also been associated with polyphenolic molecules such as flavonoids [17]. Another relevant target is the cyclooxygenase enzyme, whose inhibitors are used as anti-inflammatory agents such as salicylic acid, which gave rise to aspirin [18]. This property has also been reported to come mainly from phenolic acids and flavonoids [19].

One way to evaluate enzyme inhibitors is by high-performance thin-layer chromatography (HPTLC) coupled to biological, biochemical, and micro-chemical assays such as effect-directed analysis (EDA). This coupled system allows for the separation and detection of naturally occurring bioactive molecules in complex samples [3,15]. HPTLC-EDA ensures several benefits, such as simple sample preparation, rapid analysis of several samples at once, and unambiguous identification of the compound(s) responsible for the biological activity by using mass spectrometry (MS) [14,15,20]. In this study, the objective was to identify antioxidant molecules and inhibitors of acetylcholinesterase and cyclooxygenase enzymes present in pisco grape pomace green extracts by HPTLC-EDA-MS.

## 2. Materials and Methods

### 2.1. Reagents, Chemicals, Solvents, and Solutions

Ethanol, methanol, acetonitrile, toluene, ultrapure water, formic acid, ethyl acetate, and DPPH (2,2-Diphenyl-1-picrylhydrazyl) were purchased from Merck (Darmstadt, Germany); gallic acid, enzyme cyclooxygenase (COX-2 human; EC1.14.99.1), arachidonic acid (>98.5%), N, N, N’, N’-tetramethyl-p-phenylenediamine (TMPD; >98.5%), porcine hematin (>90%), Tris-HCl, enzyme acetylcholinesterase (AChE), Electrophorus electricus (E.C number 3.1.17), 1-naphthyl acetate, fast blue B salt, bovine serum albumin (BSA), α-glucosidase (G5300), 4-nitrophenyl-α-D-glucopiranoside (N1377), and acarbose were purchased from Sigma (St Louis, MO, USA). Diclofenac and donepezil hydrochloride (pharmaceutical secondary standard) were donated from a local pharmaceutical company.

### 2.2. Raw Material and Preparation

The pisco grape pomace was obtained from the company Pisquera de Chile S.A (Ovalle, Coquimbo Region, Chile). Once the pomace was received, it was dried in a vacuum oven [5].

### 2.3. Extract Preparation

#### 2.3.1. Extract Obtained by Enzymatic Extraction (EAE)

One gram of dry pomace was weighed and mixed with 20 mL of acetate buffer (pH 4.8; 0.1 M). The enzymes (0.75 U tannase and 40 U cellulase) were added and incubated for 15 min at 20 °C with a speed of 150 rpm (BIOBASE, Singapore, BJPX-2008). Then, 20 mL absolute ethanol was added and shaken for 30 min with a speed of 200 rpm (Boeco, Hamburg, Germany, OS-20) and centrifuged for 5 min at 4193× *g*. The extract was then frozen at −80 °C for 12 h.

#### 2.3.2. Extract Obtained by Pressurized Liquid Extraction (PLE)

One gram of dry pomace was weighed and mixed with diatomaceous earth (1:1) in a 34 mL stainless steel cell and taken for pressurized liquid extraction (Thermo Scientific Fisher, Dionex ASE 150, Hampton, NH, USA). The extraction conditions were 54% ethanol, 113 °C temperature, and three extraction cycles. The time (3 min) and pressure (103 bar) conditions were fixed. The samples were centrifuged at 4193× *g* and evaporated at 37 °C. The extract was then frozen at −80 °C for 12 h.

Both extracts were lyophilized (Friologic, Given one 5K, Santiago, Chile). The process conditions were primary drying at −20 to 20 °C with the condenser temperature set at −55 °C and secondary drying at 25 °C and a vacuum of 0.027 kPa for 24 h. The yield in both extracts was approximately 30% from the raw material.

### 2.4. High-Performance Thin-Layer Chromatography (HPTLC)

The extracts (EAE and PLE) were applied with the HPTLC (CAMAG, Muttenz, Switzerland) TLC autosampler (ATS4) on HPTLC Silica gel F254 plates using the following parameters for 8 tracks per plate: band length 8 mm, track distance 10 mm, dosage rate 50 nL/s, and the first application on the x-axis and the y-axis at 10.0 mm. Application volumes of the sample extracts were 10 µL (100 mg/mL). Chromatography was performed in a 10 × 10 cm twin-trough chamber (without saturation) up to a migration distance of 80 mm using a mobile phase consisting of ethyl acetate/toluene/methanol/water/formic acid (2.5:0.9:0.6:0.6:0.6:0.6 *v*/*v*/*v*/*v*/*v*). Both extracts were applied in duplicate on both sides of the plate to obtain two sections; the first was used for a bioassay and the second was used for mass spectrometry (MS) analysis.

### 2.5. HPTLC Bioassay for Detection of Antioxidant Molecules in Pisco Grape Pomace Extracts

Bioactive molecules present in EAE and PLE extracts were separated following the methodology described in Section 2.4. After drying the TLC plate to remove the mobile phase for 10 min at 60 °C, it was immersed into a DPPH (1% *w*/*v*) methanolic solution using a Chromatogram immersion device 3 (CAMAG, Muttenz, Switzerland).The plate was dried in the dark for 30 min, observing the antioxidant molecules as colorless bands on a purple background. Bands of 10 uL of gallic acid solution (1 mg/mL) were used as positive control.

### 2.6. HPTLC Bioassay for Detection of Acetylcholinesterase (AChE) Inhibitors in Pisco Grape Pomace Extracts

As described in Section 2.4, extracts and 10 uL of Donepezil solution (1 mg/mL; positive control) were applied on the HPTLC plate. After development, plates were dried in TLC Plate Heater 3 for 10 min at 60 °C to remove the mobile phase. After cooling to room temperature, the plates were nebulized twice with 3 mL of 100 mM Tris buffer (pH = 10) and twice with 100 mM Tris buffer (pH = 8) using a derivatizer (CAMAG, Muttenz, Switzerland) and dried at 60 °C for 10 min. The plate was then nebulized with 3 mL of the enzyme substrate 1-naphthyl acetate (1.5 mg/mL) and dried for 20 min at 60 °C. Then, 3 mL of enzyme solution (1 U/mL with BSA (1 mg/mL) in 50 mM Tris-HCl buffer at pH = 7.8) was uniformly sprayed on the plate. Finally, the plate was incubated at 37 °C for 30 min over a horizontal stand inside a closed moisture chamber containing ca. 50 mL of ultrapure water. Immediately after incubation, a freshly prepared aqueous solution of Fast Blue B salt (1.0 mg/mL) was sprayed onto the plate to obtain a purple background, contrasting with the colorless inhibition zones. The plate image was documented under white light illumination.

### 2.7. HPTLC Bioassay for Detection of Cyclooxygenase (COX-2) Inhibitors from Pisco Grape Pomace Extracts

Chromatography was carried out as described in Section 2.4. After drying the plate for 10 min at 60 °C, the plates were nebulized twice with 100 mM Tris buffer (pH = 10) and twice with 100 mM Tris buffer (pH = 8) using a derivatizer (CAMAG, Muttenz, Switzerland). After the drying step (60 °C for 10 min), plates were nebulized with the substrates (0.1 mM arachidonic acid and 50 mM TMPD) and dried for 20 min at 60 °C. Then, 3 mL of enzyme solution (0.15 U/mL COX-2 plus 0.60 μg/mL hematin in 100 mM Tris-HCl buffer, pH = 8) was uniformly sprayed onto the plate. Finally, the plate was incubated at 37 °C over a horizontal stand inside a closed moisture chamber containing ca. 50 mL of ultrapure water. After incubation, the plate was dried at 60 °C and the COX-2 inhibitors were observed as colorless bands on a purple/blue background (TMPD oxidation product). The plate image was documented under white light illumination.

### 2.8. Bioactive Compound Identification by Mass Spectrometry

Bioactive bands were cut out from the plates and analyzed by liquid chromatography (LC) mass spectrometry (MS) by means of a Waters (Milford, MA, USA) system composed of ACQUITY UPLC I-Class PLUS coupled to Synapt XS HRMS (Table 1). Separation was carried out into a BEH C18 1.7 μm 2.1 × 100 mm column (for bands 1, 2, and 3) and Phenomenex (Torrance, CA, USA) Kinetex XB-C18 3.5 μm 4.1 × 150 mm column (for band 4) using a binary mobile phase composed of water (A) and acetonitrile (B), both acidified with 0.1% (*v*/*v*) formic acid, which was used for applying the following gradient program at a flow rate of 0.4 mL/min: 0–2 min 20% B (isocratic step), 2–6 min 20–30% B, 6–14 min 30–70% B, 14–15 min 70% B (isocratic step), and 15–18 min 70–20%, plus 2 min for column conditioning for a total of 20 min per run.

Mass spectrometry analysis was performed using the following parameters: ESI (-), MS ^e^ in continuum mode, *m*/*z* range of 100–600, capillary voltage of 1.0–3.0 kV, and scan speed of 0.3 s. A collision energy ramp was set from 15 to 45 eV for high energy and 4 eV for low energy. The cone voltage was 40 V and argon was used as the collision gas. For *m/z* correction, a lock mass solution of Leucine enkephalin ([M-H]^−^ *m*/*z*: 556.2771) was applied in 30 s intervals during the sample acquisition, and mass calibration was performed using sodium formate (100−1500 *m*/*z*). Data were acquired and analyzed by Waters Masslynx software (version 4.2).

### 2.9. Characterization of EAE and PLE Extracts

The total polyphenol content of EAE and PLE extracts was determined according to the method described by Singleton et al. [21] with some modifications in a multiplate reader (Perkin-Elmer, Waltham, MA, USA, Vitor TM X3) at a 750 nm wavelength. The results were expressed in mg of gallic acid per gram of dry extract. The total flavonoid content was determined according to the method reported by Dini et al. [22] with some modifications using a UV/VIS spectrophotometer (Thermo Scientific, ORION AQUAMATE 8000, Madison, WI, USA) at 415 nm; the results were expressed in mg of quercetin per gram of dry extract. The antioxidant capacity was evaluated using different assays, namely DPPH (2,2-diphenyl-1-picrylhydrazyl) [23], ORAC (oxygen radical absorbance capacity) [24], FRAP (ferric reducing agent) [25], and ABTS (2,2′-azino-bis (3-ethylbenz-thiazoline-6-sulfonic acid)) [26]. These results were expressed in μmol trolox equivalent (TE) per gram of dry extract. In addition, the phenol profile was determined by mass spectrometry according to the conditions described in Section 2.8. UV detection was performed at 280, 320, and 360 nm.

### 2.10. Statistical Analysis

The effect of extraction methods was assessed using analysis of variance (Statgraphics Centurion XV (Statistical Graphics Corp., Herndon, VA, USA). Differences were determined to be statistically significant when *p* < 0.05. 

## 3. Results and Discussion

### 3.1. Detection of Molecules with Antioxidant Capacity in Pisco Grape Pomace Extracts

Oxidative stress plays an essential role in the development and progression of neurodegenerative diseases, and the free radical scavengers present in foods known as antioxidants are attributed to health benefits [27].

**Figure 1:**
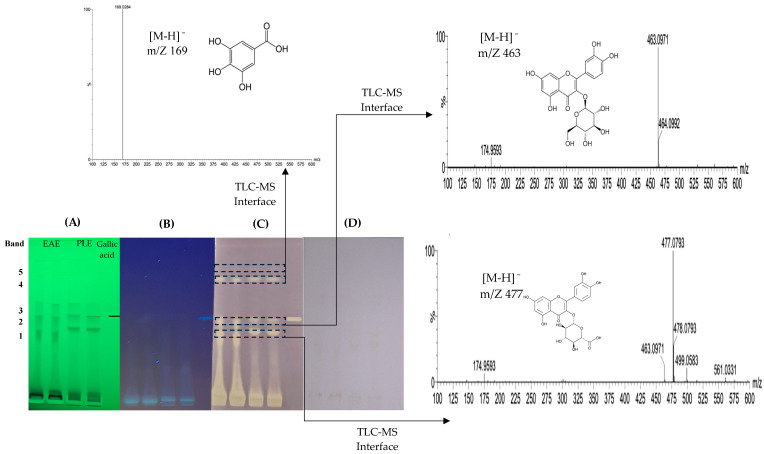
HPTLC chromatogram of pisco grape pomace extracts in TLC silica gel 60G F_254_. Detection of polyphenolic compounds with antioxidant capacity by bioassay. Photo-documentation at 366 nm, green background (**A**). Photo-documentation at 254 nm, blue background (**B**). Antioxidant capacity by DPPH, as colorless bands on a purple background (**C**). Photo documentation under white light (**D**), positive control gallic acid, and HPTLC-ESI-MS of selected bands. EAE: enzyme-assisted extraction; PLE: pressurized liquids extraction.

Figure 1 shows the HPTLC separation and the antioxidant capacity bioassay (DPPH) for the EAE and PLE extracts. The reaction of the bands with the DPPH reagent generated three yellow-colored bands representing the antioxidant compounds in both extracts. These bands demonstrate antioxidant potential by reducing the DPPH radical in the presence of an antioxidant fraction by hydrogen or electron exchange. Visually, the color of the test medium changes from purple (DPPH) to yellow (DPPH-H) [28].

Compounds present in each band were identified by mass spectrometry (Table 1). In band 1, the presence of quercetin-3-O-glucuronide was established by the presence of a parent ion at *m*/*z* 477 [M-H]^−^ and a main fragment at *m*/*z* 301. In bands 2 and 4, the compounds determined were quercetin-3-O-glucoside (*m*/*z* 463 and fragment at 300) and gallic acid (*m*/*z* 169 and fragment at 125).

Several studies have reported the presence of these compounds in different varieties of grape pomace [29,30,31,32]. Jara-Palacios et al. [33] mentioned that quercetin-3-O-glucoside is the most abundant in different types of grape pomace in Moscatel varieties, while the concentration of quercetin-3-O-glucuronide is higher in Pedro Ximénez varieties, both varieties that make up the pomace used in this work.

In the pisco pomace extracts obtained by EAE and PLE in a previous study, a concentration of 2.5 mg/g d.w. of quercetin-3-rutinoside hydrate was determined by liquid chromatography. Quercetin is a potent antioxidant due to its ability to trap free radicals and bind metal ions [34,35]; this correlates with the values obtained in the antioxidant capacity. Among the properties possessed by the identified compounds are quercetin-3-O-glucuronide, which is a primary functional flavonoid with anti-inflammatory, antiatherogenic, and antioxidant properties [36]; quercetin-3-O-glucoside exerts antioxidant, anti-inflammatory, antihypertensive, and cytoprotective activities [35]; and gallic acid is a phenolic substance widely distributed in various plants that has potent antioxidant and antimicrobial activity and is more effective in scavenging free radicals than other phenolic compounds [37,38].

**Figure 2:**
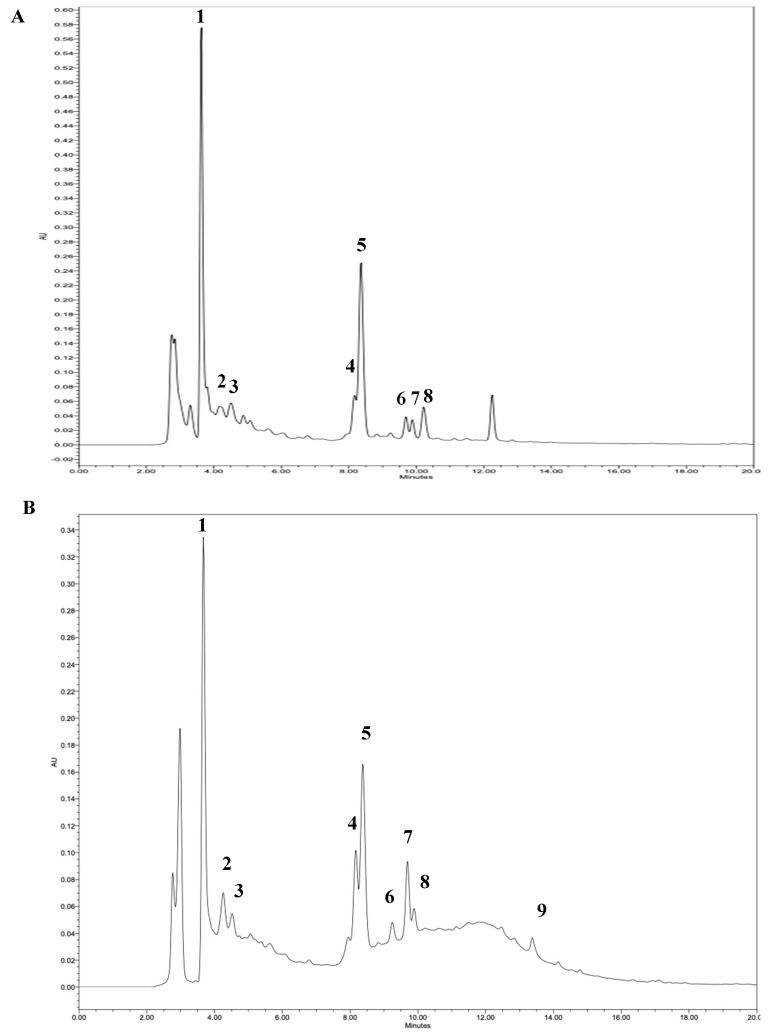
LC-MS data of phenolic compound profile of pisco grape pomace extracts. (**A**) Enzyme-assisted extraction; (**B**) extraction by pressurized liquids. (1) Gallic acid; (2) catechin; (3) (−) epicatechin; (4) quercetin-3-O-glucoside; (5) quercetin-3-O-galactoside; (6) quercetin-3-O-glucoronide; (7) quercetin-3-O-rhamnoside; (8) kaempferol-3-O-glucoside; and (9) quercetin.

These results correlate with the total polyphenol and flavonoid content in both extracts, whose values varied between 33 and 63 mg GAE/g extract and 134–254 mg QE/g extract, respectively (Table 2). The flavonoid values were higher, which is related to the compounds obtained in the phenolic profile, which mainly belong to this group of compounds (Figure 2).

The antioxidant capacity evaluated by the different assays showed that PLE obtained the highest values compared to EAE. However, both extracts showed the same biological activities. The results obtained by ORAC showed the highest values between 668 and 871 μmol TE/g extract, while between DPPH, FRAP, and ABTS, the values varied between 317 and 668 μmol TE/g extract; these variations between the assays are attributed to the mechanism of action, as they are based on redox reactions, where a reagent changes color due to its oxidative state and ability to deplete the amount of radicals [39], while ORAC uses peroxyl radicals as reagents with redox potential and a reaction mechanism similar to physiological oxidants [40] (Table 2). In addition, the IC50 of DPPH was determined, obtaining values of 1.63 ± 0.15 mg/mL extract and 1.00 ± 0.01 mg/mL extract for EAE and PLE, respectively. The IC50 was evaluated to determine the extract concentration necessary to inhibit 50% free radical scavenging; low IC50 values indicate greater antioxidant efficacy [41].

It is important to highlight that grape pomace extracts can be used in the food sector as natural food additives due to their beneficial biological properties and in the pharmaceutical industry as dietary supplements and complementary medicines, considering the content of polyphenols and how they participate in health [42].

### 3.2. Detection of Acetylcholinesterase Inhibitors in Pomace Extracts

Alzheimer’s disease (AD) is a chronic degenerative disease of the central nervous system that usually appears in older populations [43].

Acetylcholinesterase (AChE) has been used as a drug for the treatment of neurodegenerative disorders [44]. Its inhibition may be beneficial in patients with AD who suffer cognitive impairment [27].

**Figure 3:**
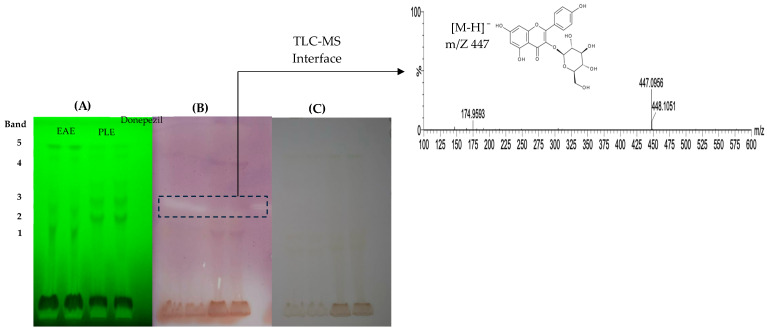
HPTLC chromatogram of pisco grape pomace extracts in TLC silica gel 60G F_254_. Detection of polyphenolic compounds inhibiting acetylcholinesterase by bioassay. Photo documentation at 366 nm, green background (**A**). Acetylcholinesterase inhibition, purple background (**B**). Photo documented under white light (**C**), positive control Donepezil, and HPLC-ESI-MS of selected bands. EAE: enzyme-assisted extraction; PLE: pressurized liquids extraction.

The AChE assay is based on the enzymatic activity converting the substrate (α-naphthyl acetate to naphthol) and its azo bonding with Fast Blue B salt (FBB) to form the purple diazonium dye (bottom of the plate) [14] (Figure 3). The HPTLC–bioassay for acetylcholinesterase inhibitor detection shows colorless bands on a purple background (EAE and PLE). MS identified the compound as kaempferol 3-O-glucoside, corresponding to band 3 with a parent ion of *m*/*z* 447 [M-H]^−^ and the main fragment at *m*/*z* 284 (Table 1). This compound has been previously reported in grape pomace [30,31,33,45]. This compound belongs to the group of flavonoids and is commonly found as glucosides, which are the most relevant. In addition, it was reported as a potential therapeutic agent because it exhibits neuroprotective activities [46,47]; the presence of this compound is related to the high values of total flavonoids obtained (Table 2).

Grape plant extracts and metabolites are an alternative in preventing and treating AD. As mentioned above, the flavonoids possess AChE inhibitory activity [48].

The HPTLC-AChE tool has been used to identify inhibitors of this enzyme in fig leaf, custard apple, sage, ginger, hemp seed oil, flax, and canola samples [3,15,16,49], but no report was found in grapes.

### 3.3. Detection of Cyclooxygenase (COX-2) Inhibitors from Pisco Grape Pomace Extracts

Selective cyclooxygenase-2 inhibitor anti-inflammatory drugs are a specific category of non-steroidal anti-inflammatory drugs designed to reduce the production of inflammatory substances [50].

Cyclooxygenase (COX-2) is involved in the inflammatory process, triggering complex physiological responses in injured tissues [20].

**Figure 4:**
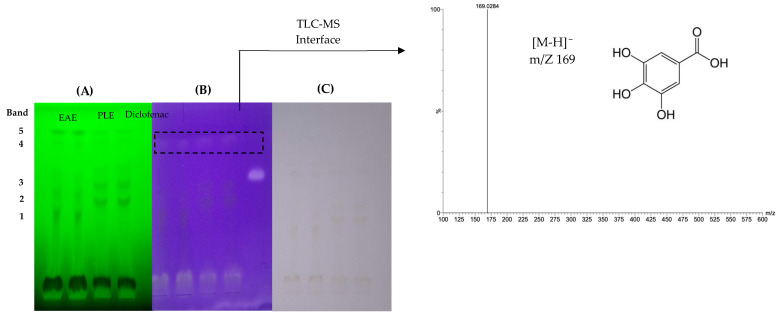
HPTLC chromatogram of pisco grape pomace extracts TLC silica gel 60G F_254_. Detection of polyphenolic compounds inhibiting cyclooxygenase by bioassay. Photo documentation at 366 nm, green background (**A**). Cyclooxygenase inhibition, purple/blue background (**B**). Photo documentation under white light (**C**), positive control Diclofenac, and HPTLC-ESI-MS of selected bands. EAE: enzyme-assisted extraction; PLE: pressurized liquids extraction.

Bands with inhibitory molecules were observed as colorless bands on a purple background (Figure 4) By MS, the COX-2 inhibitor was identified as gallic acid with a parent ion at *m*/*z* 169 [M-H]^−^ and a main fragment at *m*/*z* 125 (Table 1). This phenolic compound also exhibited antioxidant activity, as mentioned in Section 3.1. This finding is in agreement with the phenolic profile, where gallic acid was observed at 3.6 min (Figure 2). The presence of gallic acid has been reported in various types of grape pomaces [30,31,51,52,53], as well as its anti-inflammatory potential, antioxidant, and anti-diabetic agents [54,55].

The search and identification of antioxidant and inhibitory molecules beneficial for health from Pisco grape pomace obtained through green extraction techniques (EAE and PLE) can be an alternative to develop novel functional foods and, at the same time, generate a circular economy. HPTLC–bioassay–MS is a simple technique that can be used to obtain an early estimate of the biological effects of the compounds present in the extracts. The results obtained in this work reported, for the first time, molecules in pisco grape pomace with antioxidant activity, such as quercetin-3-O-glucuronide, quercetin-3-O-glucoside, and gallic acid. In addition, kaempferol-3-O-glucoside proved to be an inhibitor of acetylcholinesterase and gallic acid of cyclooxygenase. Therefore, pomace is a valuable subproduct and an agent with therapeutic potential for the food and pharmaceutical industries. In addition, these green extraction methods are sustainable processes. Although the results obtained are interesting, this study is not free of limitations. In particular, the techniques used to face scaled-up challenges make further research necessary to optimize these technological processes both at the pilot level and on an industrial scale.

## Figures and Tables

**Table 1:** Identification of phenolic compounds with antioxidant capacity and enzymatic inhibition of acetylcholinesterase and cyclooxygenase in pisco grape pomace extracts.

Band	Compounds	[M]^+^ [M-H]^−^ *m*/*z* ^a^	MS/MS Fragments *m*/*z* ^a^
1	Quercetin 3-O-glucuronide	477	301
2	Quercetin 3-O-glucoside	463	300
3	Kaempferol-3-O-glucoside	447	284
4	Gallic acid	169	125
5	Not detected	-	-

*m*/*z*: mass divided by charge number; ^a^ positive ion mode.

**Table 2:** Determination of total phenolic compounds and antioxidant capacity of EAE and PLE extracts.

	Extraction Method	
Analysis	EAE	PLE
TPC (mg GAE)/g dry extract)	33.21 ± 1.54 ^a^	62.68 ± 3.62 ^b^
TFC (mg QE/g dry extract)	134.2 ± 2.80 ^a^	254.17 ± 2.10 ^b^
DPPH (μmol TE/g dry extract)	399.4 ± 23.54 ^a^	557.61 ± 25.03 ^b^
ORAC (μmol TE/g dry extract)	668.2 ± 26.3 ^a^	871.28 ± 12.03 ^b^
FRAP (μmol TE/g dry extract)	414.4 ± 11.73 ^a^	668.22 ± 22.45 ^b^
AATS (μmol TE/g dry extract)	317.4 ± 10.67 ^a^	469.05 ± 14.65 ^b^

Values with different letters (a,b) in the same row are significantly different (*p* < 0.05). TPC: total polyphenol content; TFC: total flavonoid content; EAE: enzyme-assisted extraction; PLE: pressurized liquids extraction.

## Data Availability

The data used or analyzed during the current study are available from the corresponding author upon reasonable request.
